# Structural modulation of lithium metal-electrolyte interface with three-dimensional metallic interlayer for high-performance lithium metal batteries

**DOI:** 10.1038/srep30830

**Published:** 2016-08-03

**Authors:** Hongkyung Lee, Jongchan Song, Yun-Jung Kim, Jung-Ki Park, Hee-Tak Kim

**Affiliations:** 1 Department of Chemical and Biomolecular Engineering, Korea Advanced Institute of Science and Technology, 291 Daehak-ro, Yuseong-gu, Daejeon, 34141, Republic of Korea

## Abstract

The use of lithium (Li) metal anodes has been reconsidered because of the necessity for a higher energy density in secondary batteries. However, Li metal anodes suffer from ‘dead’ Li formation and surface deactivation which consequently form a porous layer of redundant Li aggregates. In this work, a fibrous metal felt (FMF) as a three-dimensional conductive interlayer was introduced between the separator and the Li metal anode to improve the reversibility of the Li metal anode. The FMF can facilitate charge transfer in the porous layer, rendering it electrochemically more active. In addition, the FMF acted as a robust scaffold to accommodate Li deposits compactly in its interstitial sites. The FMF-integrated Li metal (FMF/Li) electrode operated with a small polarisation even at a current density of 10 mA cm^−2^, and it exhibited a seven times longer cycle-life than that of an FMF-free Li electrode in a symmetric cell configuration. A Li metal battery (LMB) using the FMF/Li electrode and a LiFePO_4_ electrode exhibited a two-fold increase in cycling stability compared with that of a bare Li metal electrode, demonstrating the practical effectiveness of this approach for high performance LMBs.

Recently, with the growing demand for rechargeable batteries with a higher energy density for electric vehicles and utility grid applications, next-generation batteries, including lithium-oxygen (Li-O_2_) and lithium-sulfur (Li-S) batteries, have received considerable attention and have technologically evolved over the past decade^1^,^2^,^3^. Notably, the Li metal anode is a prerequisite for these kinds of batteries because it supplies the unlithiated cathode with Li^+^ and enables the design of energy-dense batteries because of its well-known superiority: high specific capacity (3,860 mAh g^−1^) and the lowest redox potential (−3.04 V) compared to a standard hydrogen electrode^4^,^5^,^6^,^7^. For that reason, the Li metal anode has once again gained wide research interest. Furthermore, the evolution of Li metal anode technology can advance lithium metal batteries (LMBs) which simply replace intercalation-based anode materials from lithium ion batteries with Li metal electrodes^8^.

However, the practical application of Li metal anodes is still limited to primary batteries because of the uncontrollable dendritic Li growth and severe surface degradation caused by the highly reactive nature of Li^6^,^9^,^10^. Such inherent problems cause the irreversible loss of Li and electrolyte, resulting in a low coulombic efficiency and even cell failure at an early stage^4^,^5^,^11^. To enhance the reversibility of Li metal electrodes, much effort has been devoted to the initial suppression of Li dendrite growth. Typical protection techniques include: (1) physical suppression by *ex-situ* coating of an artificial layer using Li^+^ion conducting polymer electrolytes^11^,^12^,^13^,^14^,^15^, a ceramic layer^16^,^17^, an inorganic-organic composite layer^7^,^18^,^19^,^20^,^21^ and a porous carbon layer^22^; (2) adoption of a highly stable electrolyte and/or the addition of an effective electrolyte additive for the *in-situ* formation of a stable solid-electrolyte interface (SEI) film^27^; (3) electrostatic control of the Li deposition behaviour by Cs^+^-salt (called a self-healing electrostatic shield, SHES)^28^,^29^.

The above strategies feature two-dimensional interface control. However, a three-dimensional architecture to confine the Li deposits can offer another effective strategy for improving the reversibility of Li metal anodes. These approaches include the manipulation of the Li dendrite growth behaviour by three-dimensional (3D) frameworks such as silica/silicon carbide (SiO_2_/SiC)-covered carbon fibre paper^30^, polyaniline/carbon nanotube (PANI/CNT) composite buffer layer^31^, nanostructured graphene framework^32^,^33^, 3D poly(acrylonitrile) fibre mat^34^, fibrous Li/B (Li_7_B_6_) matrix^35^ and hollow titanate (TiO_2_) nanotubes array^36^. For the same purpose, 3D surface modification of Li metal itself by using a micro-needle roller has been suggested^37^. The design principle, which varies depending on the 3D architecture and material used, has to date included a homogeneous Li^+^ supply, local current density reduction and spatial Li confinement.

In this study, we present a fibrous metal felt (FMF) as a 3D-conductive interlayer in order to enhance the reversibility of the Li metal electrode. The main purpose of the FMF interlayer strategy is to modulate the Li/electrolyte interfacial structure, as illustrated in Fig. 1. Typically, a bare Li metal electrode (Fig. 1a) has a propensity to form an electrochemically-inactive porous layer, which is composed of organic/inorganic compounds from the electrolyte decomposition and electrically isolated dead Li^4^,^10^,^38^,^39^. This layer can evolve in an uncontrolled manner, and expand upon cycling, enlarging the specific surface area and volume of the Li metal anode. This results in extensive consumption of fresh Li and electrolyte, the escalation of cell impedance and the abrupt fading of cell capacity. On the other hand, a pre-existing FMF interlayer in an FMF-integrated Li metal (FMF/Li) can provide the wasted dead Li particles with facile electron and ion transfer, and utilise their capacities to a higher extent (Fig. 1b). For this purpose, a stainless steel felt was employed as a proof-of-concept because a metal-based fibre network has advantageous features, such as mechanical robustness and flexibility as well as chemical and electrochemical stability^40^,^41^. Compared with polymer-, carbon- and ceramic-based 3D framework materials, a metallic framework imparts a more efficient electron transfer to the Li as well as a higher mechanical stability to endure internal stress from excessive Li metal accomodation. Furthermore, in terms of structural design, the interstitial space between the metal fibres provides a facile Li^+^ passage and a high Li accommodation capability. Indeed, the FMF-integrated Li metal electrode exhibited stable cycling at 10 mA cm^−2^ for more than 100 cycles with a notably small overvoltage of 30 mV, which has never been achieved before to the best of our knowledge. Moreover, we proved that an LMB using the FMF/Li electrode successfully operates at a 1 C-rate for more than 200 cycles.

## Results

### Lithium deposition/dissolution cycling behaviour on FMF

**Figure 2:**
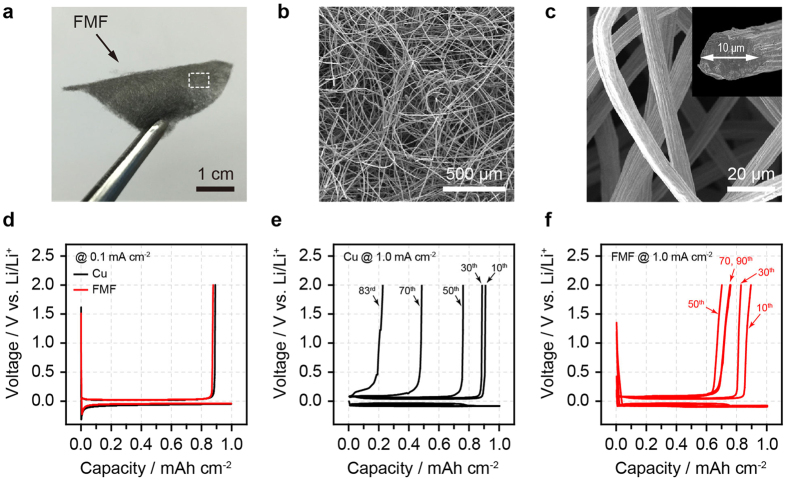
Morphology of the FMF and half-cell cycling test. (**a**) An optical image of the FMF. (**b,c**) Bird’s-eye view SEM images of the FMF with different magnifications. The inset of Fig. 2c shows the cross-section for a single stainless steel fibre. Voltage profiles of the (**d**) Cu|Li and FMF|Li half-cells at pre-cycle (I = 0.1 mA cm^−2^/Q = 1mAh cm^−2^) and those of (**e**) Cu|Li and (**f**) FMF|Li half-cells for subsequent cycling (I = 1 mA cm^−2^/Q = 1mAh cm^−2^) (10^th^, 30^th^, 50^th^, 70^th^ and 90^th^ cycle) (Cu|Li cell failed after 83 cycles).

**Figure 3:**
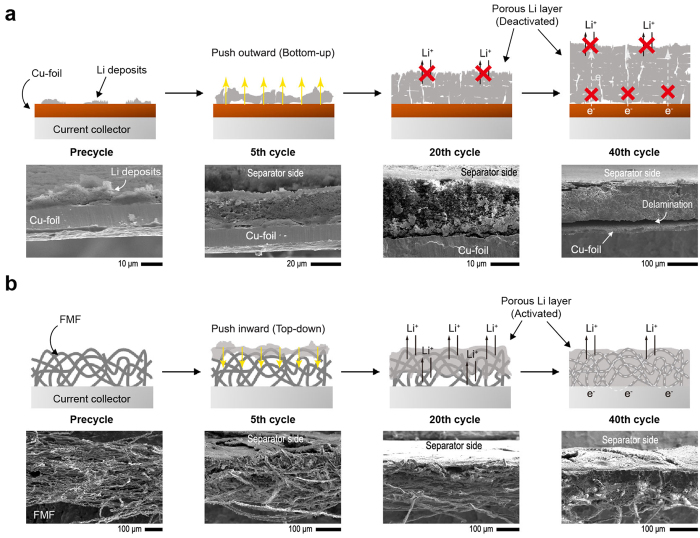
The morphological evolution of the porous Li layer on cycling. Schematic illustrations and corresponding SEM images for (**a**) Cu and (**b**) FMF electrodes during the Li half-cell cycling test.

The FMF used in this work had a fibre diameter of 10 μm, a porosity of 87% and a surface area of 0.05 m^2^ g^−1^ (Fig. 2a–c). To demonstrate the positive functions of the FMF for the reversibility of Li deposition/dissolution processes, a galvanostatic cycling test was carried out for a coin-type cell using the FMF as a working electrode and the Li metal electrode as a counter electrode, and the results were compared with those for a Cu|Li coin cell (Supplementary Fig. S1). In this half-cell configuration, Li^+^ from the Li metal electrode was electrochemically deposited (current density (I) = 1 mAh cm^−2^, capacity (Q) = 1 mAh cm^−2^) onto the Cu or FMF electrode, and then, the deposited Li was stripped away at the same current density and capacity. The coulombic efficiency (CE) can be defined as the ratio of Li removed from the working electrode to that deposited on the working electrode during the same cycle. As shown in Fig. 2d, the CEs of the first cycle were about 89% for both the Cu and FMF electrodes, which are quite close to those for carbonate-based electrolytes^6^,^23^. However, the cycle number-dependency of the CE was also quite different between the Cu and FMF electrodes, as shown in Fig. 2e,f. For the Cu electrode, the CE severely faded below 30% until 80 cycles, and the cell failed after 83 cycles. In contrast, for the FMF electrode, the CE decreased until around 50 cycles, and remained at 75% even until 90 cycles, clearly demonstrating that the introduction of the FMF enhances the reversibility of Li deposition/dissolution. The CE value of 75% for the FMF electrode is still much lower than the level required for practical applications. However, this low level is attributed to the limited stability of the 1 M LiPF_6_/EC/DEC electrolyte that was used to clearly verify the positive effect of the FMF. For a clearer understanding of the function of the FMF on Li deposition/dissolution cycling, the morphological evolution of the FMF electrode under repeated Li deposition/dissolution cycles was investigated by *ex-situ* SEM analysis. The cross sectional SEM images of the Cu and FMF electrodes after dissolution at various cycle numbers are shown in Fig. 3 and Supplementary Figs S2 and S3. For an easier understanding of the morphological evolution, corresponding illustrations are also provided in Fig. 3. These data establish three important points.

1. For Cu foil, the porous Li layer grew outward towards the electrolyte phase by generating underneath Li deposition and pushing up the former layer (Fig. 3a). In contrast, for the FMF electrode, it started from the top of the FMF and grew inward towards the current collector (Fig. 3b). Because of the facile Li^+^ transport to the porous skin of the FMF, Li deposition can initiate in the top region. After forming a Li porous surface layer (typically at 5 cycles), the subsequent Li deposition proceeds along exposed FMF fibres with lower resistance, resulting in the inward growth. This result is highly meaningful because it prevents an outward dendrite growth which often causes a short circuit. In a previous publication^30^, an inward Li growth was induced by sputtering insulating SiO_2_/SiC onto carbon fibre. However, in this work, it was simply induced by providing a more conductive framework.
2. While the porous layer developed on the planar Cu electrode eventually exhibits large cracks due to its low mechanical integrity, the FMF matrix enables the compaction of the porous Li layer to be reinforced by full-filling the interstitial spaces between the metal fibres on cycling (see Supplementary Fig. S4). For LMB application, it is worth noting that the reinforced porous Li layer can reduce the exposure of fresh Li and thus alleviate the extensive Li consumption and electrolyte decomposition at a boundary between the porous Li layer and native Li metal.
3. The weight of the porous Li layer, which corresponds to the amount of irreversible Li remaining after the 5^th^, 40^th^ and 70^th^ cycles, was lowered for the FMF electrode (see Supplementary Fig. S5a), which supports an improved reversibility. In conjunction with the electrochemical impedance spectra (Supplementary Fig. S5b), it indicates that the FMF renders the porous Li layer to be electrochemically more active by supplying a well-connected conductive matrix and this extends Li utilisation.

The half-cell test and SEM analysis collectively show that the 3D-conductive network can effectively enhance the reversibility of Li deposition/dissolution by modulating the morphology of the porous Li layer. In short, the FMF acts as an electrical conducting agent for high Li utilisation, and as a robust scaffold to confine the Li deposits compactly as well as a guide for the reversed growth of the Li deposits towards the inside of the FMF network. These characteristics had stimulated us to investigate an integrated electrode with FMF and Li metal (FMF/Li) in a Li|Li symmetric cell configuration.

### Cycling of the Li|Li symmetrical cell with the FMF/Li electrodes at high rate

**Figure 4:**
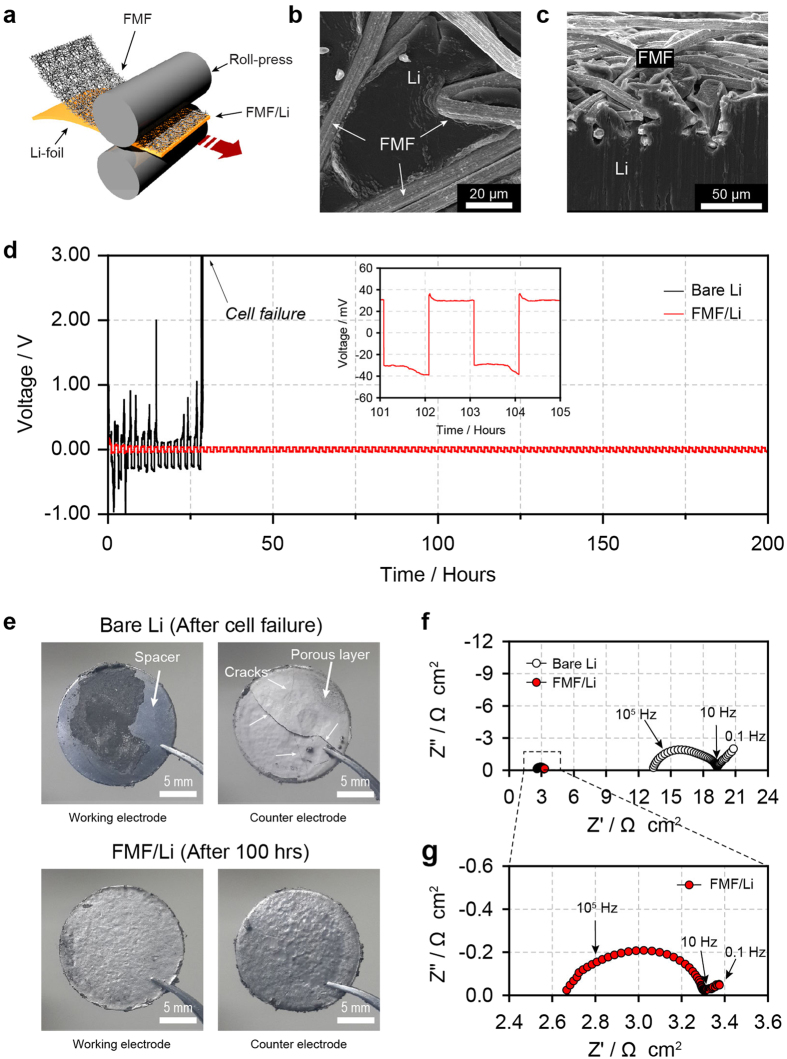
Li|Li symmetric cell test. (**a**) Schematic of the FMF/Li electrode fabrication. (**b**) Top and (**c**) cross–sectional SEM images of the FMF/Li electrode. (**d**) Time-voltage profiles of the coin-type Li|Li symmetric cells with the bare Li and FMF/Li electrodes for 100 hours at 10 mA cm^−2^ (charging/discharging = 1 h/1 h). Inset of Fig. 4d shows the magnified voltage profiles. (**e**) Digital pictures of the cell components obtained from the disassembled Li|Li symmetric cells with the bare Li electrodes and FMF/Li electrodes, respectively. (**f**) Nyquist plots of both cells using the bare Li and FMF/Li electrodes after 1 cycle; (**g**) magnified-Nyquist plots for the cell with the FMF/Li electrode.

The FMF/Li electrode was simply fabricated by roll-pressing at room temperature (Fig. 4a). Because of the high ductility of Li metal, the skin of the FMF was embedded into the Li metal electrode without any structural deformation of the FMF, as seen in Fig. 4b,c. A symmetrical Li|Li cell was used to investigate the cycling stability of the FMF/Li electrode. Supplementary Fig. S6 illustrates the symmetrical Li|Li cells with bare Li and FMF/Li electrodes. The cells were cycled at a challenging current density of 10 mA cm^−2^ with a charging/discharging time of 1 h, which corresponds to a deposition/dissolution of 48.5 μm-thick Li. As shown in Fig. 4d, the FMF/Li electrode was stably cycled over 200 h (100 cycles). In contrast, the bare Li electrodes showed increased voltage polarisations with random voltage oscillations and immediate cell failure after only 14 cycles. The sudden failure was attributed mainly to Li depletion at the working electrode, as shown in optical images of the electrodes obtained from the disassembled cell (Fig. 4e). Notably, the FMF/Li electrodes exhibited a shiny surface even after 100 cycles. The results for the Li|Li symmetric cell test is consistent with those for the Cu|Li cell test, and they clearly demonstrate that the FMF layer can improve the reversibility of Li deposition/dissolution. It should be noted here that the FMF/Li electrode had a much smaller overvoltage than that of the bare Li electrode. For the FMF/Li electrode, the overvoltage at 10 mA cm^−2^ was around 30 mV, which claims a stable Li deposition/dissolution at 10 mA cm^−2^. It is an incomparable benefit of this approach; the Li electrode polarisation can be reduced while simultaneously enhancing the CE. The extended utilisation of the Li deposits within the FMF matrix also means the expansion of the reaction surface for Li deposition/dissolution, which can explain the reduced polarisations. This is supported by the impedances of the Li|Li symmetric cell taken after the first cycle. As shown in Fig. 4g,f, the impedances are featured by a high-frequency semi-circle and low-frequency semi-circle, which correspond to the film resistance from the SEI layer formed on the Li surface and the charge transfer resistance at the interface of the SEI layer and Li, respectively. A fitting of the impedances with a circuit model (see Supplementary Fig. S7 and Table S1) shows that the charge transfer resistance of the FMF/Li is 6.4 times smaller than that of the bare Li electrode, indicating the extended utilisation of the porous Li layer and the three-dimensional expansion of the redox reaction surface. The film resistance, which is inversely proportional to the active reaction surface, was also 11.5 times smaller for the FMF/Li, supporting the expansion of the reaction surface by the FMF. Moreover, the Warburg resistance, which is indicative of Li^+^ diffusion though the porous layer to a reaction surface, was 28.4 times smaller for the FMF/Li than for the bare Li. This behaviour can also be explained by the high electrochemical activity of the porous layer. For the bare Li, Li^+^ should pass through the thick inactive porous layer for the redox reactions, resulting in a large diffusional resistance. By contrast, for the FMF/Li electrode, the diffusion length is significantly shortened because the redox reaction can happen within the porous layer.

### Electrochemical performance of the LMB with the FMF interlayer

**Figure 5:**
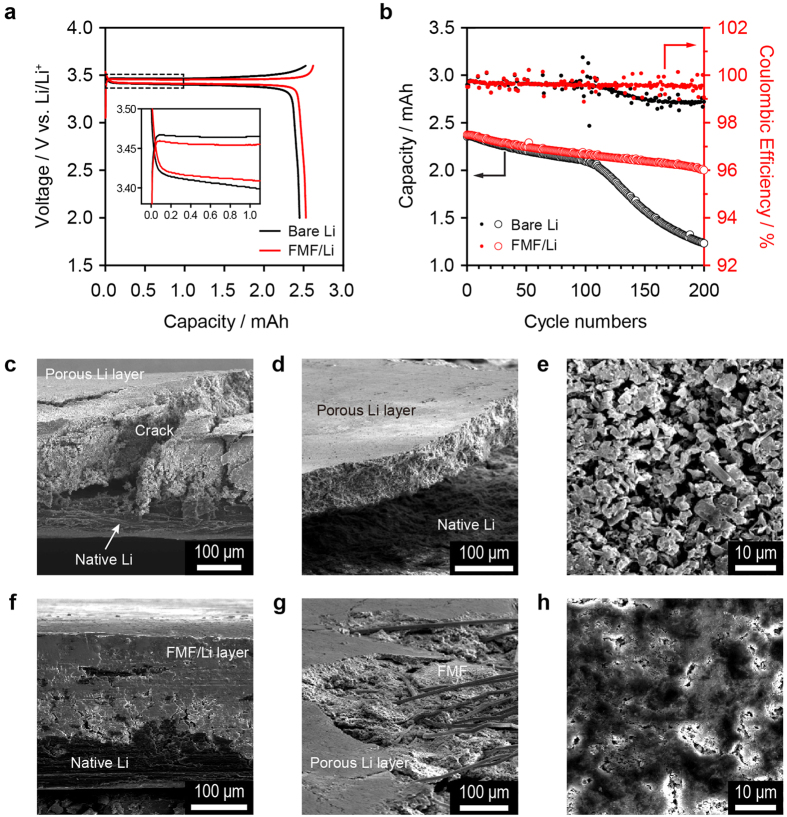
Cycling of Li|LFP cells with and without FMF interlayer. (**a**) Charge-discharge profiles of the Li|LFP cells using the bare Li and FMF/Li electrodes measured at a 0.1 C-rate (0.164 mA cm^−2^). Inset shows the magnified potential curves. (**b**) Cycling performances and coulombic efficiencies at a 1 C-rate (1.64 mA cm^−2^). SEM images of cross-sectional, tilt (15 °) and top views of the bare Li (**c–e**, respectively) and FMF/Li (**f–h**, respectively) electrodes after 200 cycles of the Li|LFP cells at a rate of 1.64 mA cm^−2^ (1 C-rate).

As an assessment of the applicability of FMF to LMBs, coin-type Li|LiFePO_4_ (LFP) cells were fabricated, and their battery characteristics and cycling stability were measured. Cell configuration is illustrated in Supplementary Fig. S8. The LFP cathode had an areal capacity of 1.64 mAh cm^−2^, and thus, the 1 C-rate corresponds to a current density of 1.64 mA cm^−2^. Figure 5a shows the charge and discharge voltage profiles during pre-cycling at 0.1 C for the bare Li and FMF/Li electrodes. Because of the reduced polarisation of the FMF/Li electrode, the charge and discharge overvoltages were smaller, and the discharge capacity was larger than those for the bare Li. More interestingly, as shown in Fig. 5b, the FMF/Li cell exhibited a considerably improved cycling performance compared with the bare Li cell. The FMF/Li cell retained a capacity retention of 86.4% and a coulombic efficiency of 99.5% after 200 cycles. In contrast, the bare Li cell significantly faded after 115 cycles, revealing a capacity retention of 86.0% and a coulombic efficiency of 99.0% after 122 cycles.

The SEM images of the Li electrodes taken from the Li|LFP cells after 200 cycles are presented in Fig. 5c–h. For the bare Li, the separation of a 180 μm-thick porous Li layer with cracks from the original Li electrode was identified. The poor contact between the porous Li layer and original Li electrode would lower the utilisation of the porous Li layer. For the FMF/Li, the electrodeposited Li was compactly packed into the FMF layer, and the Li-filled FMF tightly adhered to the original Li electrode (see Fig. 5f,g). These images clearly confirm the formation of an FMF-embedded Li layer with cycling. Additionally, the surface of the FMF/Li electrode did not indicate any dendritic Li growth, supporting the inward growth of Li.

## Discussion

The motivation behind the ‘3D-conductive interlayer’ approach emerges from our finding that the sudden capacity fading of LMBs originated from Li surface deactivation by the development of a highly resistive porous Li layer. This detrimental behaviour was observed regardless of Li metal electrode thickness. When we carried out the cycling test of Li|LFP cells with 150 and 450 μm-thick Li anodes at a 1 C-rate, each cell started to fail at the 120^th^ and 140^th^ cycles, respectively (see Supplementary Fig. S9). Despite a three-fold abundance of Li resource, there is no significant improvement on the cell’s cycle life. Considering the excess amount of electrolyte, we believe that capacity fading of LMBs is directly related to the surface failure of the Li metal anode, which is attributed to the tremendous growth of the highly resistive porous Li layer. This observation can also support the recent study by D. Lu *et al*. which suggested that the origin of cell failure owes to a tremendous escalation of cell resistance from the growth of a porous Li layer^4^.

In this regard, our focus is differentiated from previous works related to the design of 3D-structured current collectors. For example, Yang *et al*. reported advanced porous architectures accommodating Li metal inside 3D Cu foil with a submicron skeleton^42^. Compared to such elaborate nano-engineering, the FMF used in this study is commercially available, and building the FMF interlayer on Li foil is very feasible to boost the cell performances in a practical manner. A composite electrode is readily useable as the anode material in advanced Li battery systems including unlithiated high-capacity cathode materials (e.g. S and O_2_), without any pre-lithiation process. A 3D-conductive interlayer can consequently be applied to various types of next-generation Li batteries.

**Figure 6:**
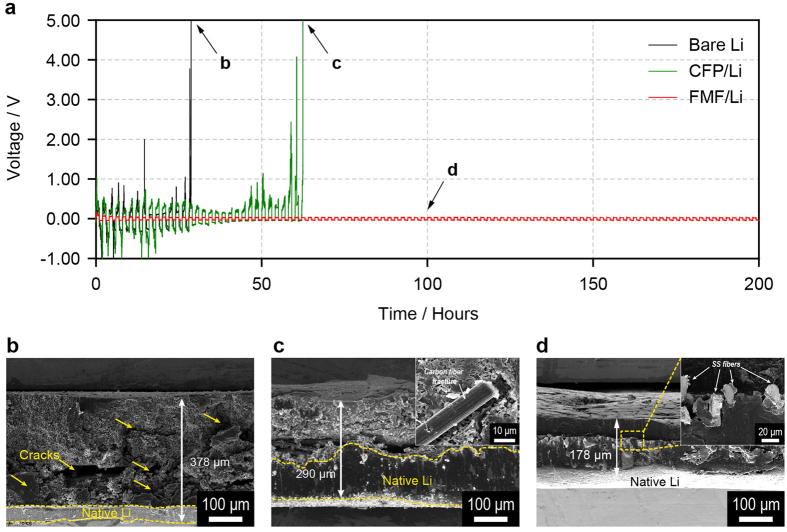
Importance of mechanical stability of 3D-conductive interlayer. (**a**) Time-voltage profiles of the coin-type Li|Li symmetric cells with the bare Li, CFP/Li and FMF/Li electrodes for 200 hours at 10 mA cm^−2^ (charging/discharging = 1 h/1 h). SEM images obtained from the disassembled Li|Li symmetric cells; (**b**) bare Li and (**c**) CFP/Li after cell failure and, (**d**) FMF/Li after 100 hours. In Fig. 6b, the yellow arrows indicate the large cracks in the porous Li layer, resulting from its poor integrity. Inset of Fig. 6c shows the carbon fibre fracture observed at the surface region of CFP/Li electrode. Inset of Fig. 6d presents the SS fibres embedded in the cycled FMF/Li electrode.

In the design of the 3D-conductive interlayer, the internal stress generated by the expansion of the porous Li layer should be considered. According to the *ex-situ* SEM analysis for the Cu|Li cell (Fig. 3), the thickness of the porous Li layer increased quickly in the first 30 cycles, and the thickness reached approximately 175 μm until the 70^th^ cycle. Likewise, a large expansion of the Li metal anode inevitably generates a compressive stress in the clamped cell, which is geometrically confined by a metal case. Although compressive stress is known to be beneficial for suppressing the growth of a porous Li layer^43^, it can cause mechanical degradation of the 3D-conductive interlayer. This problem can be exemplified by a carbon fibre paper (CFP) interlayer of which thickness (~120 μm) and fibre diameter (10 μm) are similar to those of the FMF. Even though the CFP and FMF both have similar architectures and a highly conductive nature, the Li|Li symmetric cell employing the CFP/Li electrode exhibited an imcomparably poorer Li reversibility (31 cycles) compared to that of the FMF/Li electrode, as shown in Fig. 6a. In order to account for the difference in reversiblity between the CFP/Li and FMF/Li electrode, the SEM images for the bare Li, CFP/Li and FMF/Li after cycling were collected (Fig. 6b–d, respectively). As expected, the thickness of bare Li significantly increased from 150 μm to 378 μm until cell failure due to excessive growth of porous Li layer (Fig. 6b). Although the overalll thickness of the CFP/Li electrode measured after the cell failure (~290 μm) was relatively smaller than that of the bare Li, the CFP-embedded porous Li layer was delaminated from the native Li metal, and further, the carbon fibres were fractured as evidenced by the SEM image of the surface of of the porous Li layer (the inset of Fig. 6c). Based on these observations, the limited reversibility of the CFP/Li electrode is due to the mechanical deterioration of the CFP interlayer during the cycling. By contrast, the mechanical integrity of the FMF/Li electrode was well-maintained without delamination, suppressing the volume expansion of Li metal (Fig. 6d). These results imply that the FMF can present excellent mechancial robustness and withstand compressive stress, whereas CFP could not afford to accomodate the excessive growth of the porous Li layer due to its inherent brittleness.

The mechanical property of the 3D interlayer is also important for cell design and fabrication. For the application of a 3D-conductive interlayer to winding type or flexible batteries, the interlayer should be flexible and have a high bending tolerance. A bending test was conducted for the CFP and FMF to highlight the merit of the metal-based 3D interlayer compared to the carbon fibre-based one (Supplementary Fig. S10). For the CFP, 180° bending with a radius of curvature of 4.23 mm led to a resistance rise above the detection limit, which is inadequate for winding cell manufacturing and flexible battery applications. By constrast, the metallic interlayer withstands 180° bending and even folding, preserving its high conductance. Owing to its intrinsic mechanical robustness and flexibility, the metallic interlayer is highly robust under the porous layer growth and any bending/folding deformations.

Despite the advantages of the FMF, achieving a high energy density in LMBs employing a current 3D-conductive interlayer may be criticised due to the high density of stainless steel (8.03 g cm^−3^). However, given the high conductivity, mechanical robustness and flexibility of the FMF, the FMF can function as a current collector, allowing the Li metal electrode to eliminate the conventional Cu foil current collector that is required even for the design of practical lithium metal batteries^8^. Indeed, the areal density of the FMF used in this work is equivalent to that of 11 μm-thick Cu foil. Furthermore, it is noteworthy that the preservation of the initial energy density on long-term cycling is of paramount importance as well as a densification of the battery. As depicted in Supplementary Fig. S11, the cell with the FMF interlayer can reduce energy density loss (54% → 26% for gravimetric, and 70% → 42% for volumetric), and its specific energy densities after 200 cycles surpass those of the control cells as part of the improved cell performance and the reduced volume expansion of Li metal. Therefore, even though the FMF interlayer is inactive towards lithium, resulting in a disadvantage with respect to specific energy densities, its unique benefits provided by the metallic 3D-conducting interlayer far exceed the disadvantages, and it is beneficial to overall performance. Further, this study can be extended for the development of advanced metallic 3D interlayers with a lighter and thinner metallic material or a hollow fibre-type material in order to deliver the practical energy densities.

In summary, the 3D-conducting network of FMFs can improve the reversibility of Li deposition/dissolution at high current densities with small polarisations. The porous Li layer grows inward from the top of the FMF, eliminating the problem of dendrite-induced short circuits. The densely occupied Li within the FMF framework shows a reduced irreversible Li formation and polarisation. It was shown with a Li|Li symmetric cell test that the FMF/Li electrode can operate at a high current density of 10 mA cm^−2^, with a small polarisation of 30 mV. Importantly, the excellent mechanical stability of FMF can permit a permanent electrical conduction network to confine Li deposits densely without structural damage and local isolation of the electron transfer path. Furthermore, the proto-type LMB based on an LFP cathode and FMF/Li with a practical active material loading has a cycling stability of more than 200 cycles at a 1 C-rate. This approach thereby offers a new potential possibility for improving the cycling stability of a Li metal anode by building a microstructured interlayer with a robust electrical conducting network.

## Methods

### Fabrication of the FMF-integrated Li metal (FMF/Li) electrode

Stainless-steel fibre felt (Sfelt^®^ Jenax Co. Ltd, South Korea) was used. The FMF was pressed by a roll-press to a thickness of 100–120 μm. The FMF and metallic Li foil (thickness: 0.15 mm), which was purchased from Honzo, Japan, were sandwiched by roll-press lamination in an Ar-filled glove box. As-prepared FMF/Li was cut into an electrode with a diameter of 16 mm.

### Cell assembly and electrochemical measurements

For all electrochemical measurements, 2032 coin-type cells were assembled in an Ar-filled glove box consisting of the electrodes, a polypropylene separator (PP2400, Celgard, USA) and an electrolyte (1 M LiPF_6_ in EC/DEC (50/50 by volume, PANAX ETEC Co. Ltd, South Korea)). Galvanostatic experiments were performed using a battery cycler (WBCS 3000, WonAtech, South Korea) at 25 °C.

### Cu|Li half-cell test

For the Cu|Li cell construction, 20 μm-thick Cu foil and FMF were used as the working electrode, and a 450 μm-thick Li disc was used as the counter and reference electrodes. Cells containing the electrodes, separator and electrolyte were assembled in an Ar-filled glove box. To exclude the electrolyte depletion effect, an excess amount of the electrolyte (200 μl) was used. Prior to the cycling test, all cells were pre-cycled at a rate of 0.1 mA cm^−2^ for 10 hours. Cycling tests were carried out by first depositing 1 mAh cm^−2^ of Li onto the Cu electrode, followed by Li dissolution up to 2 V.

### Li|Li symmetric cell test

For the Li/Li symmetric cell test, 2032 coin-type cells were constructed with the same configuration, excluding the substitution of the working electrode to the Li electrode. Prior to the cycling test, all cells underwent a pre-formation step at a rate of 1 mA cm^−2^ for 10 cycles (charging/discharging = 5 h/5 h). Electrochemical impedance spectroscopy measurements were obtained with a Solartron 1255 (Solartron Analytical, UK) frequency response analyzer (FRA) together with a Solartron 1287 electrochemical interface over a frequency range of 1 MHz to 0.1 Hz.

### Li|LiFePO_4_ (LFP) cell test

For the Li|LFP cell test, carbon-coated LFP (LFP/C) powder (~5 wt.% of carbon) was used as received from Hanwha Chemical Co. Ltd, South Korea. The cathode was prepared by casting a slurry consisting of 90 wt.% LFP/C, 5 wt.% Super-P carbon black (Timcal, Switzerland), 5 wt.% polyvinylidene fluoride (PVdF) (Sigma-Aldrich, USA) binder and *N*-Methyl-2-pyrrolidone (NMP) as a solvent onto an Al current collector. After the slurry was dispersed, the cathodes were dried at 120 °C for 24 h. The mass loading density and the thickness of the electrode were controlled to 11.82 mg cm^−2^ and 56 μm (except from the current collector), respectively. Thus, the electrode density was 2.1 g cm^−3^. As a negative electrode, a pristine Li metal disc (150 μm- and 450 μm-thick) or a FMF/Li metal electrode was used. In order to examine the performance of the Li|LFP cell, the assembled cells were cycled at 0.164, 1.64 mA cm^− 2^ with a voltage cut-off of 3.6 V and 2.0 V for charging and discharging, respectively.

### Characterisation

The Li metal electrodes were carefully separated from the disassembled cells after electrochemical cycling. To remove any residual electrolyte, the electrodes were rinsed with anhydrous dimethyl carbonate (DMC, Sigma-Aldrich, USA) and subsequently dried under vacuum at room temperature for 24 h. Next, SEM holders with as-prepared Li metal samples were hermetically sealed inside an air-tight polypropylene bottle (Nalgene, 60 mL) for safe transfer without contamination. The procedure, including sealing, was executed in an Ar-filled glove box. Immediately before the SEM measurement, all samples were coated with platinum (Pt) in a coater (SCD005, Bal-Tec, 30 mA, 120 sec) to avoid charging effect. After that, the Pt-coated samples were quickly transferred to the SEM analysing chamber. To comparatively examine the surface morphologies of the Li metal electrodes, FE-SEM (Sirion, FEI) images were obtained. SEM was undertaken with an acceleration voltage of 10 kV and a working distance of 5.0 mm.

## Additional Information

**How to cite this article**: Lee, H. *et al*. Structural modulation of lithium metal-electrolyte interface with three-dimensional metallic interlayer for high-performance lithium metal batteries. *Sci. Rep*. **6**, 30830; doi: 10.1038/srep30830 (2016).

## Supplementary Material

Supplementary Information

## Figures and Tables

**Figure 1:**
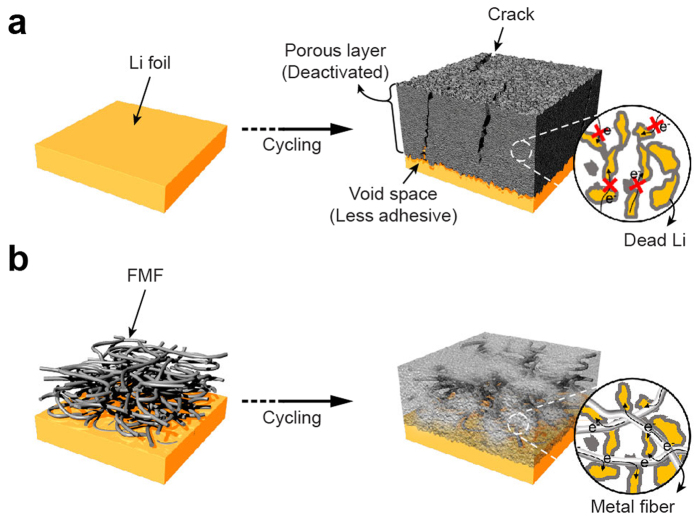
Concept of building a 3D-conductive interlayer. Schematic diagrams of the evolution of the porous Li layer for (**a**) bare Li and (**b**) FMF/Li.
