# Supplementary material for: Structural modulation of lithium metal-electrolyte interface with three-dimensional metallic interlayer for high-performance lithium metal batteries
Source: Sci Rep. 2016 Aug 3;6:30830. doi: 10.1038/srep30830 (PMC4971473; doi:10.1038/srep30830)
Supplement: Supplementary Information [file srep30830-s1.pdf]

Supplementary Information

for

Structural modulation of lithium metal-electrolyte interface with three-dimensional metallic interlayer for high-performance lithium metal batteries

Hongkyung Lee, Jongchan Song, Yun-Jung Kim, Jung-Ki Park & Hee-Tak Kim*

Supplementary Figures

Supplementary Figure S1 -- **S2**

Supplementary Figure S2 -- **S3**

Supplementary Figure S3 -- **S4**

Supplementary Figure S4 -- **S5**

Supplementary Figure S5 -- **S5**

Supplementary Figure S6 -- **S6**

Supplementary Figure S7 -- **S6**

Supplementary Figure S8 -- **S7**

Supplementary Figure S9 -- **S7**

Supplementary Figure S10 -- **S8**

Supplementary Figure S11 -- **S9**

Supplementary Tables

Supplementary Table S1 -- **S10**

Supplementary Table S2-- **S11**

Reference

Supplementary Figures

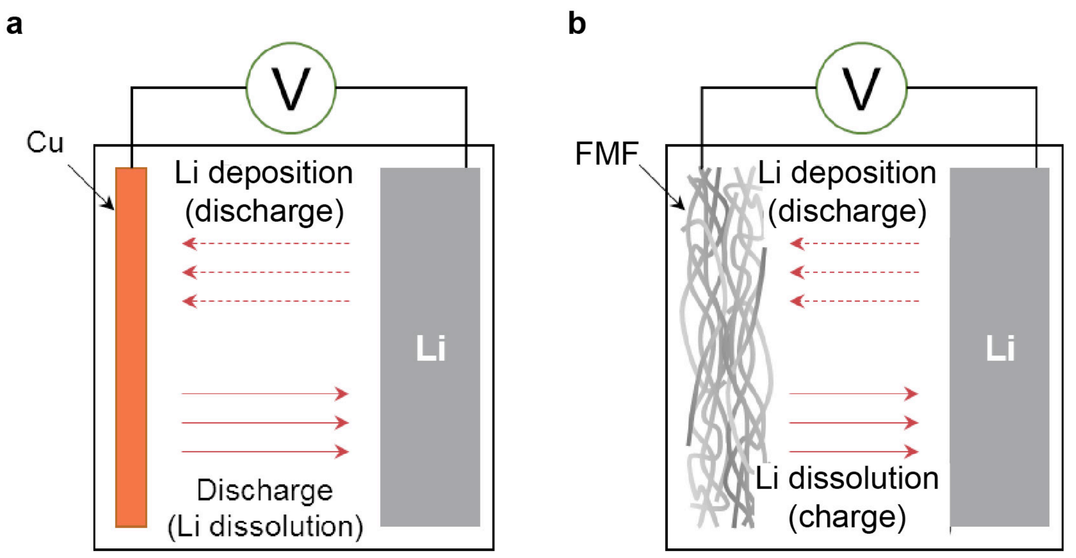

Supplementary Figure S1 | Schematic illustrations for half-cell configurations. (a) Cu|Li and (**b)** FMF|Li cells, respectively.

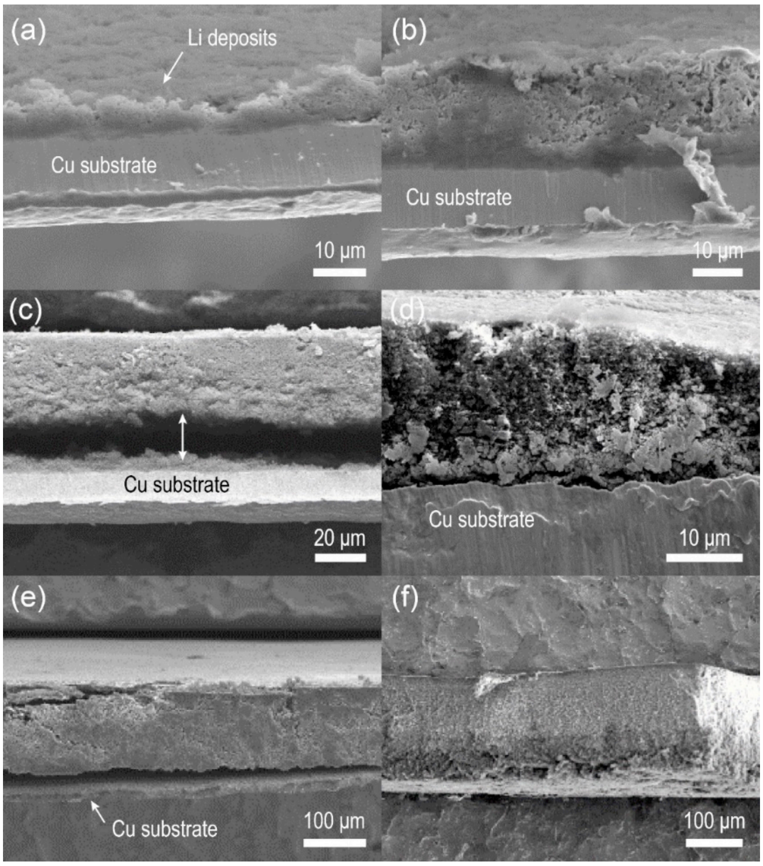

Supplementary Figure S2 | Cross-sectional morphological evolution of the porous Li layer on the Cu electrode during Li deposition/dissolution cycle; at (**a**) precycle, (**b**) fifth, (**c**) tenth, (**d**) twentieth, (**e**) fortieth, and (**f**) seventieth cycle.

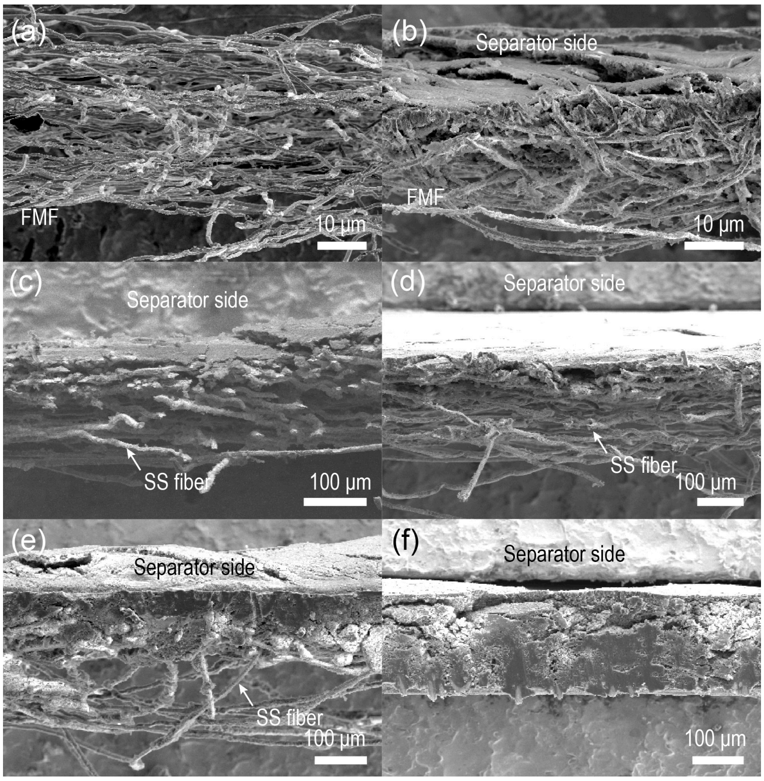

Supplementary Figure S3 | Cross-sectional morphological evolution of the porous Li layer on the FMF electrode during Li deposition/dissolution cycle; at (**a**) precycle, (**b**) fifth, (**c**) tenth, (**d**) twentieth, (**e**) fortieth, and (**f**) seventieth cycle.

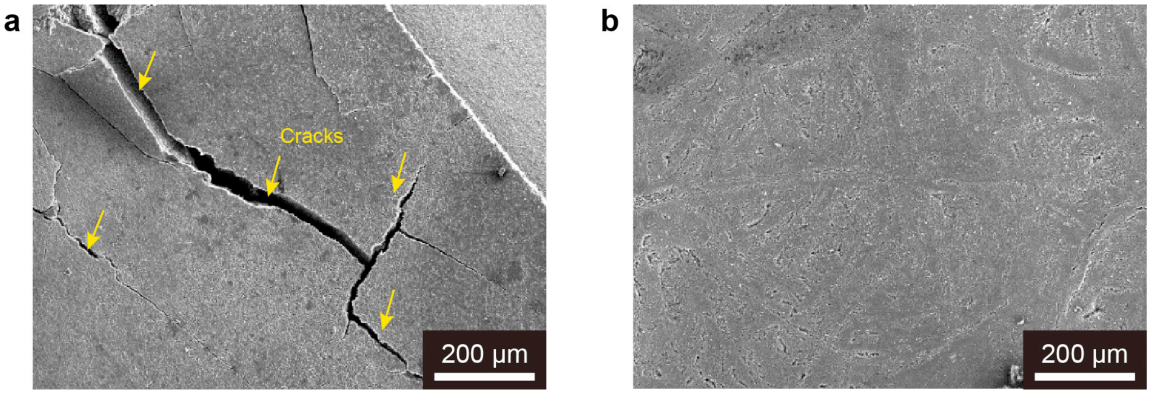

Supplementary Figure S4 | Bird-eye view SEM images for the porous Li layer obtained from half-cells after 40 cycles; (**a**) Cu electrode and (**b**) FMF electrode

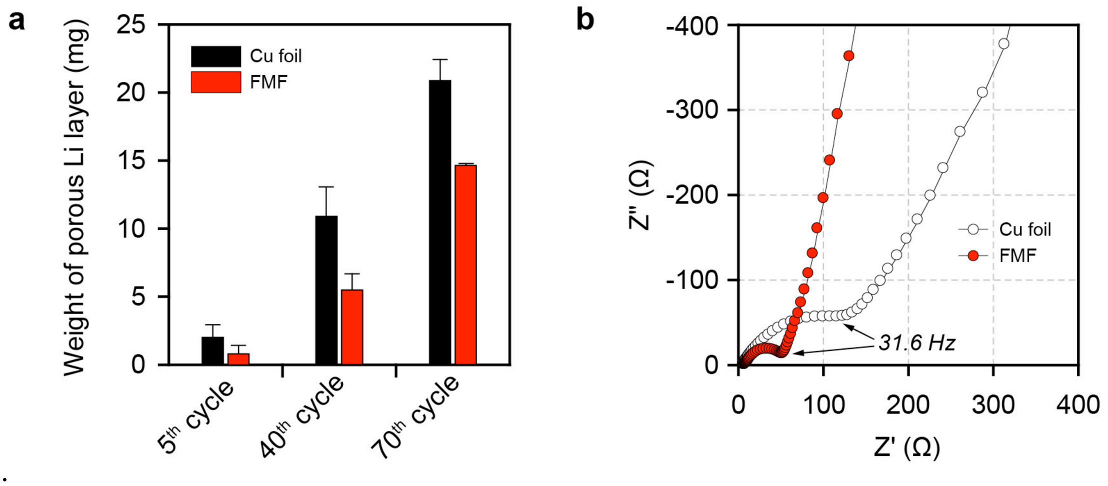

Supplementary Figure S5 | Measurement of the weight and the resistance of electrodes after half-cell cycling; (**a**) quantitative measurement of the weight of porous Li layer on Cu and FMF electrodes at selected cycles. Prior to measurement, the mass of Cu and FMF electrodes were measured beforehand to determine the weight of porous Li layers only. All values are means of triplicate analyses. (**b**) Electrochemical impedance spectra (EIS) for Cu|Li and FMF|Li half-cells at open-circuit voltage (OCV) after 40 cycles.

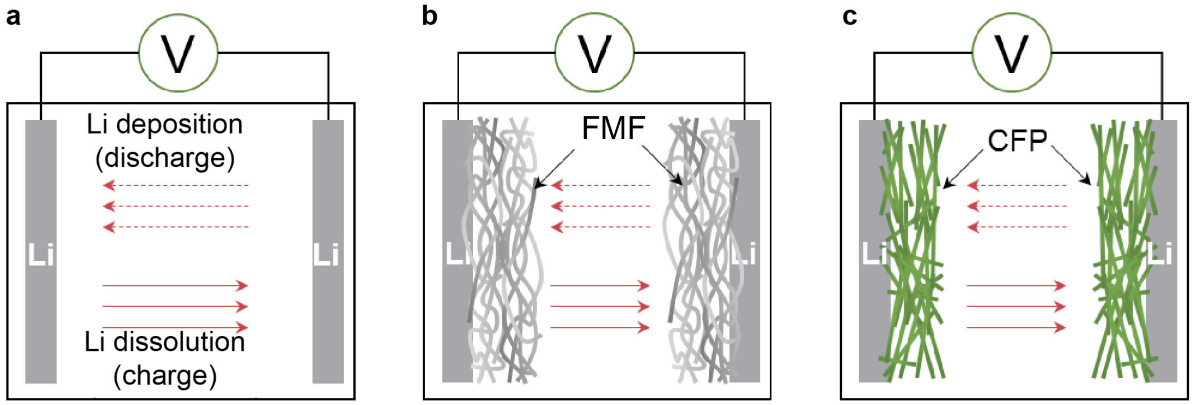

Supplementary Figure S6 | Schematic illustrations for Li|Li symmetric cell configurations. (**a**) bare Li, (**b**) FMF/Li, and (**c**) CFP/Li electrodes

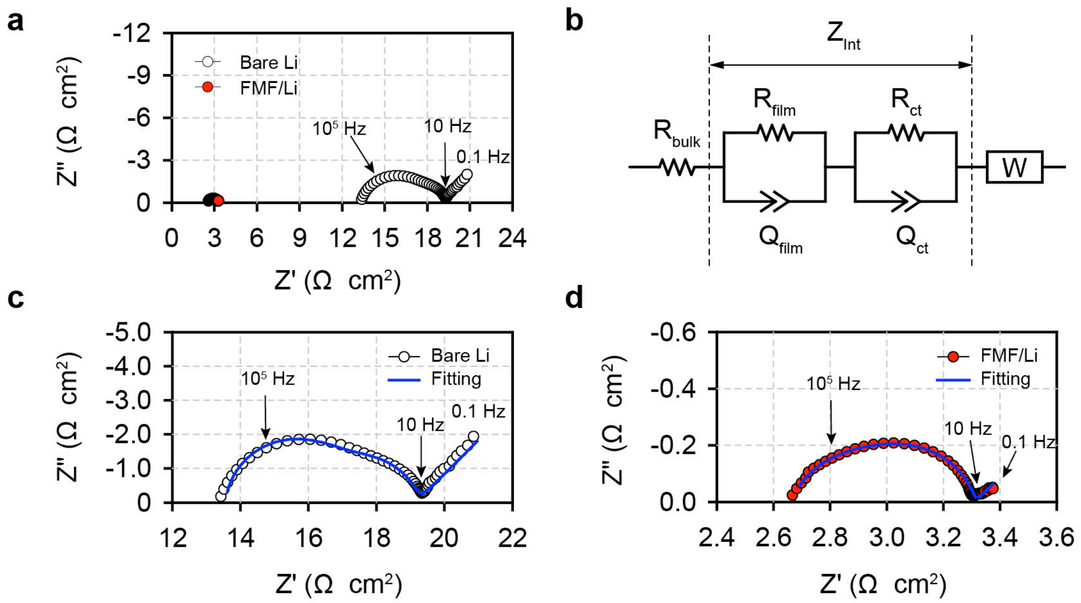

Supplementary Figure S7 | EIS analysis of the Li|Li symmetric cells with and without the FMF interlayer at a current density of 10 mA cm^{-2}. Each cycle lifetime lasts 1 h. (**a**) Nyquist plots of the two symmetrical cells employing bare Li and FMF/Li after 1 cycle. (**b**) The equivalent circuit model was used for the fitting of the impedance data. Fitting results for (**c**) the bare Li and (**d**) FMF/Li, respectively.

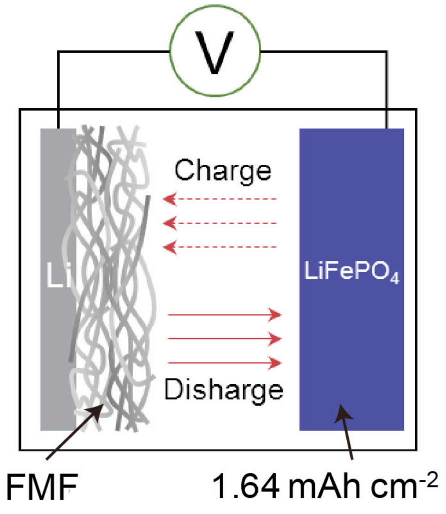

Supplementary Figure S8 | Schematic illustrations for Li|LiFePO$_4$ (LFP) cell configuration.

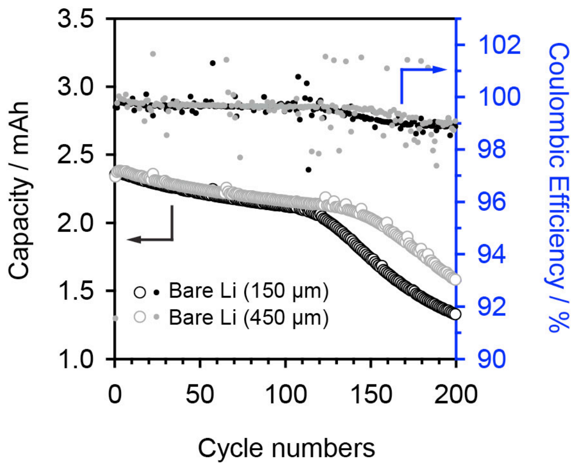

Supplementary Figure S9 | Cycling performances and Coulombic efficiencies of Li|LFP cells with two bare Li electrodes with different thickness (Li thickness: 150 and 450 μm) at 1 C-rate (1.64 mA cm^{-2}).

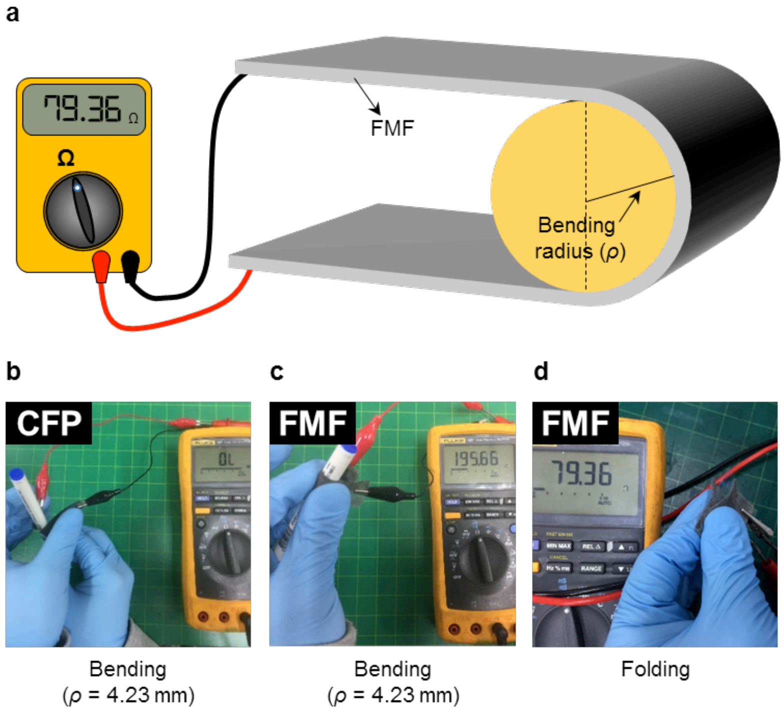

Supplementary Figure S10 | Examination of the flexibility of 3D conductive interlayers. (**a**) Schematic illustration for bending test. Measurement of resistances for curved (**b**) CFP and (**c**) FMF sheets (2 cm width, 6 cm length) with bending radius, 4.23 mm. (**d**) Resistance value of fullly folded FMF sheet.

Through bending test, we simply examine the flexibility of 3D conductive interlayer. Generally, elementary bending mechanics in sheet-type materials, where the bending strains are determined by the film thickness, t, divided by twice the bending radius, ρ.[S1] Thus, the smaller the bending radius, the greater is the material flexibility. With 4.23 mm of bending radius, the CFP sheet was broken out, showing overlimited resistance (OL in resistance meter display, see Supplementary Fig. S10b), whereas the resistance of FMF sheet was well-maintained under same curvature. Even more, the fully folded FMF sheet still allowed the electrical conduction, showing relatively lower value of resistance.

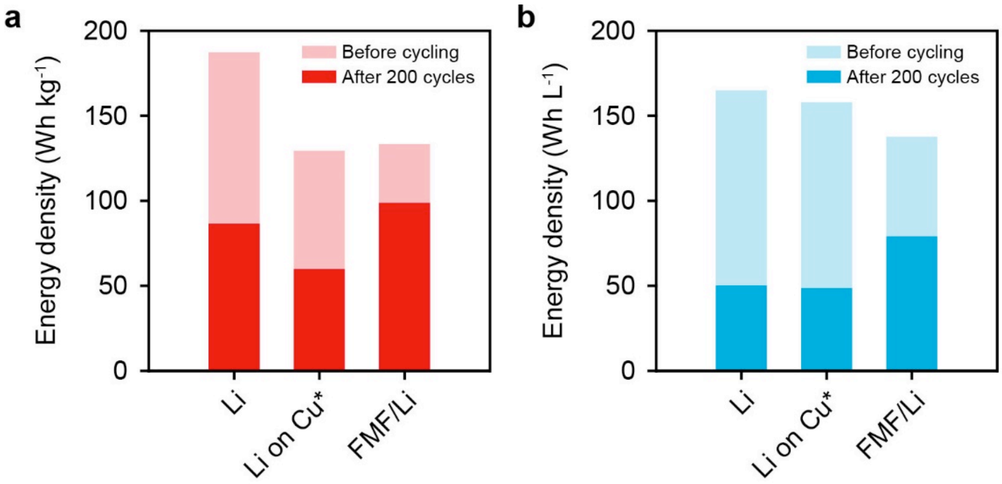

Supplementary Figure S11 | Calculated energy density on a coin cell base. (**a**) gravimetric and (**b**) volumetric energy densities of Li|LFP cells before and after cycling. For this calculation, the weight of the coin cell casing and the electrolyte was excluded. Detailed information about the cell specification is displayed in Supplementary Table S2.

Supplementary Tables

Supplementary Table S1 | List of equivalent circuit model parameters determined from the circuit model for the impedance data (Supplementary Fig. S7c and d) of the Li|Li symmetrical cells with and without the FMF interlayer.

	R_{bulk} /Ω·cm^2	R_{film} /Ω·cm^2	Q_{film} /F·cm^2 s$^{(\alpha 1-1)}$	α_1	R_{ct} /Ω·cm^2	Q_{ct} /F·cm^2 s$^{(\alpha 2-1)}$	α_2	W
Bare	13.6	3.34	18.8×10^{-6}	0.953	2.27	0.467×10^{-3}	0.869	1.42
FMF/Li	2.66	0.290	1040	0.788	0.357	1.99	0.851	0.05

An EIS analysis provides a more quantitative description of the role of the FMF interlayer as a well-connected conductive matrix. The impedance data were described with a circuit model (Supplementary Fig. S7b) which features the bulk resistance (R_{bulk}), interfacial film resistance ($R_{int} = R_{film} + R_{ct}$) which is the sum of interfacial film resistance (R_{film}) and charge transfer resistance (R_{ct}), interfacial capacitance of the constant phase element (CPE) (Q_{int}), and the Warburg diffusion element (W). In order to reflect the inhomogeneity of the Li electrode interface, CPE is used instead of the ideal capacitance. By fitting the circuit model to the data, the circuit model was well-fitted to both EIS spectra, and the obtained parameters are presented in Supplementary Table S1. Especially, it is noteworthy that the charge transfer resistance within the porous Li layer was significantly reduced by the FMF matrix (2.271 → 0.357 Ω·cm^2).

Supplementary Table S2 | Cell information and energy density calculation based on total weight and volume of the cell components. [1)] For the Li on Cu electrode, the values were calculated assuming that it shows identical performances with Li metal. [2)] The areal mass loading of FMF was ~10 mg cm^{-2} and, the total thickness of FMF/Li electrode after roll-pressing was approximately 210 μm.

Cell specifications

Cell component	Parameters		Only Li	Li/Cu[1)]	FMF/Li[2)]
Cu current collector (diameter: 16 mm)	Thickness (μm)		-	11	-
	Weight (mg)		-	19.72	-
Li anode (diameter: 16 mm)	Thickness (μm)	Before cycling	150	150	210
		After 200 cycles	280	280	300
	Weight (mg)	Before cycling	16.1	16.1	36.2
		After 200 cycles	-	-	-
Separator (diameter: 18 mm)	Thickness (μm)		26	26	26
	Weight (mg)		3.68	3.68	3.68
$LiFePO_4$/C cathode (diameter: 14 mm)	Thickness (μm)		56	56	56
	Weight (mg)		11.82	11.82	11.82
Al current collector (diameter: 14 mm)	Thickness (μm)		30	30	30
	Weight (mg)		12.46	12.46	12.46

Energy density calculation

Total weight (mg)		44.02	63.73	64.11
Total volume (cm^3)	Before cycling	0.0403	0.0420	0.0495
	After 200 cycles	0.0603	0.0620	0.0634
Capacity (Ah)	Before cycling	0.00245	0.00245	0.00253
	After 200 cycles	0.00132	0.00132	0.00205
Nominal Voltage (V)	Before cycling	3.366	3.366	3.378
	After 200 cycles	2.886	2.886	3.093
**Gravimetric energy density (Wh kg^{-1})**	**Before cycling**	**187.2**	**129.3**	**133.2**
	**After 200 cycles**	**86.5**	**59.77**	**98.8**
**Volumetric energy density (Wh L^{-1})**	**Before cycling**	**164.8**	**157.8**	**137.6**
	**After 200 cycles**	**50.1**	**48.6**	**79.0**

Reference

S1. Kim, D.-H. *et al.,* Stretchable and foldable silicon integrated circuits. *Science* **320**, 507-511 (2008).
